# A Role for Folate in Microbiome-Linked Control of Autoimmunity

**DOI:** 10.1155/2021/9998200

**Published:** 2021-05-19

**Authors:** Christine Mölzer, Heather M. Wilson, Lucia Kuffova, John V. Forrester

**Affiliations:** ^1^University of Aberdeen, Institute of Medical Sciences, Aberdeen AB25 2ZD, UK; ^2^Eye Clinic, Aberdeen Royal Infirmary, Aberdeen, UK

## Abstract

The microbiome exerts considerable control over immune homeostasis and influences susceptibility to autoimmune and autoinflammatory disease (AD/AID) such as inflammatory bowel disease (IBD), multiple sclerosis (MS), type 1 diabetes (T1D), psoriasis, and uveitis. In part, this is due to direct effects of the microbiome on gastrointestinal (GI) physiology and nutrient transport, but also to indirect effects on immunoregulatory controls, including induction and stabilization of T regulatory cells (*T*_reg_). Secreted bacterial metabolites such as short-chain fatty acids (SCFA) are under intense investigation as mediators of these effects. In contrast, folate (vitamin B9), an essential micronutrient, has attracted less attention, possibly because it exerts global physiological effects which are difficult to differentiate from specific effects on the immune system. Here, we review the role of folate in AD/AID with some emphasis on sight-threatening autoimmune uveitis. Since folate is required for the generation and maintenance of *T*_reg__,_ we propose that one mechanism for microbiome-based control of AD/AID is via folate-dependent induction of GI tract *T*_reg__,_ particularly colonic *T*_reg_, via anergic T cells (*T*_an_). Hence, folate supplementation has potential prophylactic and/or therapeutic benefit in AID/AD.

## 1. Introduction

Autoimmune diseases (AD) develop when there is breakdown of immunological tolerance to self-antigen in the adaptive immune system while autoinflammatory diseases (AID) occur when there are defects or dysregulation in the innate immune system [1]. In both cases, a disordered microbiome has been implicated and, by inference, an altered bacterial flora including its secreted products [2]. Classical AD such as multiple sclerosis (MS) [3], type 1 diabetes (T1D) [4], and rheumatoid arthritis (RA) [5] is kept at bay by a healthy microbiome, while probable AID such as inflammatory bowel disease (IBD), Behçet's uveitis, and ankylosing spondylitis (AS) are negatively affected by a disordered microbiome (reviewed in [6]). Psoriasis, a debilitating skin inflammation, and uveitis, a major sight-threatening disease in which infection may be a direct or indirect cause, are considered in many cases to be either an AD or an AID [7, 8].

In both AD and AID, there is failure of immune regulation (tolerance) and a disturbed microbiome. Identifying possible causal links between these two biological domains is a major focus. In adaptive immunity, tolerance (homeostasis) is maintained by autoreactive T cell deletion/anergy or suppression by T regulatory cells (*T*_reg_). *T*_reg_ are also effective in controlling innate immunity by regulating the activity of myeloid and NK cells [9] and so contribute to preventing AID. Circumstantial evidence for their role in AD and AID is the decline in *T*_reg_ numbers in many of these conditions such as AS [10] as well as the effectiveness of adoptive *T*_reg_ therapy in experimental models of AD and AID.

## 2. The Colonic Microbiota Shapes the Host's Health

The prenatal GI tract is sterile due to the protective immunological placental barrier preventing bacterial translocation into the fetal organism. Microbial colonialization develops gradually when environmental contact first occurs upon delivery. This has significant implications for overall health in later life [11, 12]. For instance, the expanding gut microbiome exerts its effects on brain- (CNS-) related immune privilege (IP) in that the blood-CNS barriers only reach maturity in the neonatal period [13–15]. A key colonic metabolite that can modulate the immune system is folate. Naturally, occurring folate/vitamin B9 (pteroyl-glutamic acids and oligo-glutamic acid conjugates) and its synthetic form folic acid (FA) are water-soluble B vitamins that must be ingested through the diet (e.g., legumes and leafy greens [16, 17]) or supplements [18]. Commensal bacteria [19] are also capable of synthesizing folate and other B vitamins. Glutamic compounds [20] occur in the body as different metabolites with variable bioavailability [21] and the terms folate and FA are often used interchangeably. The role of folate in hematopoiesis, reproductive health and foetal development are well known, and an extended role for the vitamin particularly in later life is recognized in preventing a decline in cognitive and neurological functioning [16, 22, 23]. Indeed, most likely due to inadequate intake, folate deficiency is more prevalent in the older population [24] contemporaneously with a higher incidence of chronic disease.

Hence, a balanced microbiome with adequate folate and micronutrient production maintains homeostasis. Recently, however, the microbiome has come under scrutiny as a source of pathogenic antigens capable of inducing or promoting AD [25–28]. This is particularly linked to dysbiosis [29] and may be the result of infection with pathogenic bacteria, loss of commensal bacteria, or reduction in microbial diversity [30]. The human intestinal epithelium covers as much as 400 m^2^ of surface area [31] with more than ten times as many resident microbes as the total number of cells in the body [32]. Overall, the gut microbiota comprises five phyla and about 160 species in the large intestine [33], and the number of genes of the intestinal microbiota is 150 times greater than the human genome [34]. Qualitative and quantitative changes in the microbial flora, their metabolic activity, and their local distribution [35] are a typical feature of IBD [36] that is otherwise characterized by the infiltration of the lamina propria with a mixed leukocyte population expressing proinflammatory cytokines [37]. Whether dysbiosis represents the cause or result of IBD (reviewed in [38]), it is a correlated biomarker of extraintestinal inflammatory disease (reviewed in [25, 39]). While mechanistic evidence is still limited, dysbiosis has long been linked to AD [40], including noninfectious uveitis [41–43], (reviewed in [25]), often occurring simultaneously with acute flare-ups of colitis [44]. Thus, it can be seen that dysbiosis and similar microbiota-related environmental factors impact up to 70% of all AD [45, 46], and while the etiology of IBD itself is not fully understood, it is considered to be the result of an interplay between environment/nutrition, microbiota, gastrointestinal immunity, and epigenetics.

## 3. The Microbiome Promotes Immunological Tolerance via an Immune Privilege-Like Mechanism

Immune privilege is a relative property of all tissues reflecting various degrees of tissue-based immunological tolerance [47] and has particular relevance for the large intestine, now considered a secondary immune organ [48, 49]. “Unconventional” IP of the gut [50] tolerates trillions of commensals and has two components, a physicochemical barrier and an immunological barrier [51]. The physical barrier is provided by the two cellular barriers which prevent translocation of pathobionts from the intestine to the general circulation (reviewed in [47]). These include a monolayer of enterocyte epithelium (i.e., an intestinal epithelial barrier, IEB) covering the entire mucosa and the subjacent lamina propria and a stringent gut-vascular barrier (GVB). The physical barrier to the passage of small molecules is provided by immunologically responsive [50] intraepithelial tight junctions [52] while a chemical barrier derives from specialized enterocytes (mucus-producing goblet cells and Paneth cells) which secrete antimicrobial peptides (reviewed in [53, 54]). A further physical barrier to hematogenous passage of any pathogens which may have penetrated the epithelium is provided by the GVB [55] with its closely associated pericytes and enteric glial cells which serve a vital function in retaining barrier properties [56–60].

The immunological component of the gut barrier is provided by a wealth of immune cells in the gut, including tolerogenic DC, several types of classical T cells including *T*_reg_, three sets of innate lymphoid cells (ILC), myeloid suppressor cells, and mucosa-associated invariant T (MAIT) cells. These cells regulate aspects of both the adaptive and innate immune systems and are under the control of secreted factors both by host cells and the microbiome. For instance, flagellin associated mostly with gram-negative bacteria (e.g., *E.coli* and *Salmonella*) binds TLR5 on CD103^+^ mucosal DC which secrete IL23 to act on ILC which in turn release IL22 to then induce the gut epithelium to release antimicrobial peptides [30], and colonic *Clostridia* through their metabolic activity have been found to induce and impact the colonic distribution of *T*_reg_ in mice [61–63]. *T*_reg_ are known to be stabilized by folate [64–66], that in turn is synthesized by some commensals including *Clostridia*, *Lactobacilli*, and *Bifidobacteria* [67]. These findings point to a tolerizing immunological role for the commensal microbiome that has the potential to exert effects on disease induction and progression. How gut-derived leukocytes might cross distant barriers at target sites and induce AD/AID remains to be clarified. Several mechanisms have been proposed [25] viewed from both the perspective of an adaptive immune response (TCR activation) and dysregulation of the microbiome.

## 4. The Microbiome Mediates Immunological Tolerance via the Products of Microbial Fermentation

**Figure 1:**
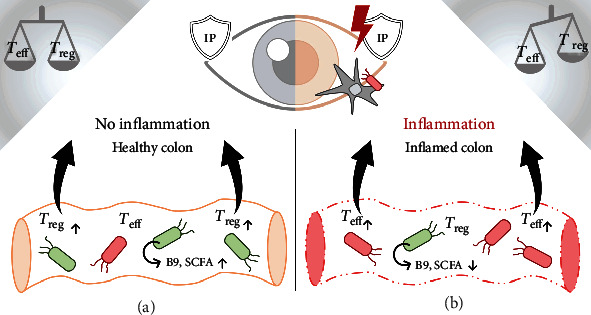
Bioavailable folate (B9) produced by certain phyla of the human microbiome (*Proteobacteria*, *Firmicutes*, *Actinobacteria*, and *Verrucomicrobia* [84]; green microbes) and short-chain fatty acids (SCFA) locally stabilize T regulatory cells (*T*_reg_) in the colon, thereby increasing their abundance (a). T cells traffic from the colon to distant sites [85, 86] where they accumulate and exert their respective functional properties. The inflamed colon ((b); characterized by structural damage—“leaky gut”) is frequently accompanied by dysbiosis (red microbes). This qualitative and quantitative shift in bacterial colonization is associated with decreased microbial folate and SCFA production and a consequential relative increase in autoreactive immunogenic T effector cells (*T*_eff_) [87]. *T*_eff_ have been shown to traffic from the colon to target sites of autoimmunity [85, 86] (e.g., intraocular tissue in the case of autoimmune uveitis), skewing the ratio of immunogenic (*T*_eff_) to regulatory cells (*T*_reg_) at target sites [88–90], ultimately breaching ocular immune privilege (IP) through unknown mechanisms, and thereby triggering autoimmune disease. Due to impaired intestinal barrier integrity in dysbiosis, pathogenic bacterial/viral/fungal/environmental antigens have facilitated access to the circulation, possibly triggering inflammation through adjuvant effects at affected sites (following antigen presentation; APC) (b). In the case of uveitis, we propose that when a sufficiently high *T*_eff_ precursor frequency is generated [91], activated T cells access retinal tissue where they adopt a pathogenic phenotype upon further activation by retinal self-antigen and/or microbial antigen [92].

Microbial fermentation in the gut generates secreted products which directly modify immune activity (Figure 1). These include products of tryptophan metabolism, short-chain fatty acids (SCFA), and folate.

### 4.1. Tryptophan

Tryptophan is an essential amino acid (AA) that is delivered through the diet, particularly dairy products and fish. Host metabolic pathways of tryptophan include the serotonin and kynurenine routes, the latter of which via indoleamine 2,3 dioxygenase (IDO) is a major tolerizing pathway in DC and macrophages. Its downstream products such as kynurenic acid (KA), 3-hydroxy-anthranilic acid (HAA), quinolinic acid (QA), and niacin (vitamin B3) suppress both innate and adaptive immunity and promote immunological tolerance and gut homeostasis (reviewed by [68]). Tryptophan can also be metabolized by microbiota-generating metabolites that interact with the aryl hydrocarbon receptor [69]. The IDO pathway and the AhR system are active in many cell types and important in homeostasis, e.g., in epithelial health. In immune cells, it mediates tolerance and suppresses inflammation via DC-mediated induction of *T*_reg_.

### 4.2. Retinoic Acid

Induction of *T*_reg_ in the gut may also require supplementation of dietary vitamin A (retinol) which is directly converted to bioavailable all *trans*-retinoic acid (atRA) by gut-associated lymphoid tissue DC [70] and in both mice and humans promotes conversion of naïve T cells into tissue-specific (mucosa homing) FoxP3^+^*T*_reg_ through FoxP3 promoter histone acetylation [71]. Moreover, atRA prevents the IL6-induced conversion of *T*_reg_ into Th17 cells and boosts the generation of TGF*β*-induced *T*_reg_ in vitro that were effective in suppressing inflammation in a colitis model [72]. Similarly, atRA stabilized *T*_reg_ in an experimental autoimmune encephalitis model (EAE) through a TGF*β*-dependent pathway [73], and in an experimental autoimmune uveitis (EAU) model, atRA acted as an adjuvant to induce antigen-specific type 1 *T*_reg_ (Tr1) attenuating autoimmunity [74].

### 4.3. SCFA

SCFA produced by the microbiome are major effectors of immunomodulation. Three main SCFA are recognized: acetate, butyrate, and propionate. Acetate accounts for ~50-70%, propionate ~20%, and butyrate, which is selectively restricted to certain *Clostridia* species, makes up the remainder [75]. SCFA regulates intestinal *T*_reg_ and macrophages, and the majority of SCFA produced remain in the gut generating beneficial effects locally, while only negligible quantities escape into the general circulation [76]. Dysbiosis in IBD (colitis) patients is typically associated with a reduced number of bacteria that produce SCFA particularly butyrate and propionate (Figure 1(b)). These include *Firmicutes* such as cluster IV *Clostridia* next to *Bifidobacteria*. Propionate and butyrate suppress inflammation by promoting the generation of tolDC and *T*_reg_ (reviewed in [77]). In germ-free mice treated with antibiotics, stool butyrate concentrations were decreased relative to littermate mice [78]. Oral acetate supplementation in NOD mice reduced autoreactive T cells in lymphoid tissues in a B cell-mediated fashion. A butyrate-rich diet increased *T*_reg_, while a combination of both SCFA improved gut barrier function and decreased diabetogenic IL21 in serum of NOD mice [79]. Colonic concentrations of SCFA, including butyrate, correlated with the number of FoxP3^+^*T*_reg_ in the caecum of mice [62]. Furthermore, oral administration of butyrate to mice increased the FoxP3 expression in *T*_reg_, higher numbers of *T*_reg_ in mucosal tissues, and an enhanced ability of DC to induce *T*_reg_ differentiation. These data suggest that butyrate (and to a lesser extent propionate) promotes extrathymic differentiation of *T*_reg_ [78]. Recent findings from human trials investigating AD (MS and neuromyelitis optica) support this hypothesis [80, 81].

Vitamin B3 (niacin) also exerts immune-modulatory functions by increasing *T*_reg_ cell numbers and functioning [82, 83] together with butyrate through activation of its receptor Gpr109a, thereby protecting against colon inflammation [83].

Mechanistically, SCFA act by inhibiting histone deacetylases (HDAC) [78], in antigen-presenting cells affecting atRA and IL10 production [93]. HDAC also induce apoptosis in *T*_eff_ cells [94] and engage in loosening of chromatin, thus enabling transcription factor accessibility to the DNA backbone. These findings suggest a balancing effect of SCFA on mucosal and systemic immunity, possibly affecting inflammation in secondary organs, since mucosal inflammation is typically associated with epithelial damage (“leaky gut”). Fukuda et al. [95] demonstrated that acetate (produced by *Bifidobacteria* and *Clostridia*) may improve leaky gut by restoring gut epithelial integrity through activation of the inflammasome and IL18 [96].

### 4.4. Folate

**Figure 2:**
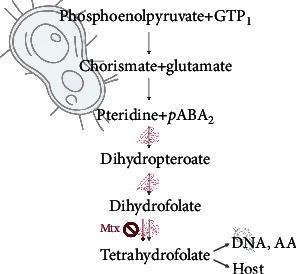
Bacterial route of de novo folic acid synthesis. Certain commensals of the human colonic flora produce folate de novo through the chorismate pathway (from phosphoenolpyruvate + guanosine triphosphate, GTP)_1_. Further, 77% of the bacterial genome is capable of synthesizing folate using freely available *p-*aminobenzoic acid (*p*ABA) and dihydropteroate diphosphate (pteridine)_2_ [84]. The pathway engages a series of enzymatic reactions (red protein symbols) including dihydropteroate synthetase and dihydrofolate synthetase. The resulting dihydrofolate (via the dihydropteroate intermediate) must be enzymatically reduced (through dihydrofolate reductase) to generate biologically active tetrahydrofolate. This process can be blocked by the folate antagonist methotrexate (Mtx), used for controlling some forms of autoimmune disease including anterior uveitis. Folate metabolites synthesized by commensals are used by the bacteria themselves (e.g., for DNA-synthesis or anabolic pathways such as generation of amino acids, AA). The remaining unused folates are released into the gut lumen and absorbed in a receptor-mediated fashion absorption into the circulation.

Folate is synthesized de novo from phosphoenolpyruvate and guanosine triphosphate (GTP) and secreted by commensals of the phylum *Bacteroidetes* (*Prevotella*, *Bacteroides*, *Porphyromonas* [97]) (reviewed in [98]) (Figure 2). In contrast to dietary vitamins that are mostly absorbed in the small intestine, microbial folate metabolites are mainly absorbed in the colon where they are produced [99, 100] and assimilated into host tissues [100–102]. Various glutamylation profiles for commensal gut microbes (i.e., species-specific patterns of folate derivatives) may affect folate bioavailability in the intestine [84] and the general circulation [67], and the colon is recognized as a significant folate depot [19]. Folic acid deficiency is associated with disruptions of intestinal integrity and persistent diarrhea (reviewed in [103]). A folate-producing microbiome likely influences the T cell methylome, but the mechanism is unclear [94]. Rats fed a probiotic formulation of folate-producing *Bifidobacteria* exhibited increased plasma folate levels, confirming in vivo production and absorption of the vitamin. The same supplement when administered to humans raised folate concentration in feces (reviewed in [67]). A sufficient folate status therefore is likely to reduce the risk of AD/AID including uveitis.

## 5. Microbiome-Mediated Immune Tolerance Is Maintained through Regulatory and Anergic T Cells under the Influence of Folate

Deletion, anergy, and induction of *T*_reg_ are the tenets of immune tolerance. *T*_reg_ are generated centrally in the thymus de novo (natural *T*_reg_, n*T*_reg_) and in the periphery (p*T*_reg_) from conventional T cells (*T*_conv_). Most autoreactive *T*_conv_ are deleted in the thymus or periphery but a proportion may enter a state of anergy (*T*_an_) as they become tolerized [104–107]. p*T*_reg_ in the gut are recognized as major contributors to immune homeostasis and generated in response to tryptophan metabolites or SCFA secreted by the microbiome.

Less is known about the role of microbiome-generated folate in colonic *T*_reg_ formation. However, an important property of p*T*_reg_ is the high expression of the folate receptor, FR4 [65]. In addition, a developmental relationship between *T*_reg_ and *T*_an_ has been suggested [105, 106, 108, 109]. Both cell types have some overlapping features such as expression of the folate receptor FR4 [108]. Moreover, on adoptive transfer, anergic FoxP3^−^ CD44^hi^ CD73^hi^ FR4^hi^ Nrp1^+^ cells gave rise to FoxP3^+^*T*_reg_ in an autoimmune arthritis model and reduced the susceptibility of mice to IBD [110], through acting as progenitors for *T*_reg_ cell differentiation. Both *T*_reg_ and *T*_an_ rely on similar tightly regulated epigenetic programs to retain function [105, 109, 110]. n*T*_reg_ contain highly methylated CpG-rich regions in the conserved noncoding sequence 2 (CNS2) of the FoxP3 locus ([111, 112]; reviewed in [113]) since p*T*_reg_ are induced in the periphery, this level of methylation is lost allowing stable FoxP3 expression (summarized in [114]). Thus, systemic folate may be more important in the generation of n*T*_reg_ rather than stable p*T*_reg_ as exist in the colon. However, the increased number of total methylation sites in *T*_an_ in the periphery [115] probably allows a necessary degree of instability to permit interconversion of *T*_an_ and p*T*_reg_. This points towards a potential indirect *T*_reg_ replenishing effect of folate through epigenetic modifications in *T*_an_. Microbiome-derived folate might thus generate a pool of *T*_an_ from *T*_conv_, which have the option of losing their methylation sites and becoming stable *T*_reg_. This degree of flexibility underpins the properties of immunological tolerance.

The mechanism whereby folate modifies *T*_reg_ appears to be through inhibiting cell death specifically by induction of Bcl-2. Adoptive transfer of *T*_reg_-depleted cell suspensions induced autoimmune gastritis in susceptible nude mice [65] while adoptive transfer of folate-supplemented *T*_reg_ prolonged the cells' survival and protected the mice from the disease. Mice treated with the folate antagonist methotrexate (Mtx) show impaired survival of *T*_reg_ and decreased expression of Bcl-2, while in vivo depletion of dietary folate resulted in a reduction in *T*_reg_ cell numbers in the small intestine. In this study, folate was required for the survival of differentiated n*T*_reg_, but not for the conversion of naïve T cells into p*T*_reg_ [66]. Remarkably, this effect is different from that of atRA, and less so vitamin D3, which both enhance the differentiation of naïve T cells into p*T*_reg_ [72, 116–119], emphasizing a unique role for folate in the generation of n*T*_reg_ [113]. This selective effect of folate on maintenance of FoxP3^+^*T*_reg_ has been further demonstrated [64], while a diet deficient in folate resulted in a marked reduction of FoxP3^+^*T*_reg_, but not other T cell populations, in the colon. In the same study, blockade of FR4 and treatment with Mtx, led to decreased colonic FoxP3^+^*T*_reg_ and increased autoimmune bowel inflammation. These data have implications for human biology but remain to be verified in man, particularly as *T*_reg_ exhibit some degree of phenotypical variation between mice and humans [120, 121].

## 6. FoxP3 in T Regulatory Cells Controls the Expression of the Folate Receptor

**Figure 3:**
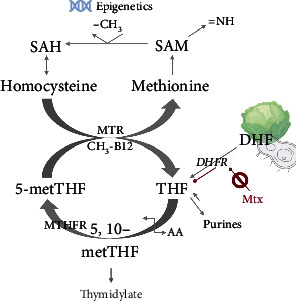
Pathways of folate metabolism and the interrelationships of folate-dependent reactions. Dihydrofolate (DHF) from nutritional sources and the gut microflora is enzymatically reduced engaging dihydrofolate reductase (DHFR) to biologically active tetrahydrofolate (THF), a process that is competitively blocked by the folate analogue methotrexate (Mtx). This has implications for cell proliferation, division, and survival. Folate metabolism branches out into anabolic pathways including synthesis of amino acids (AA) and amines (=NH) as well as purines and thymidylate for DNA production. Importantly, folate in the form of 5-methyl tetrahydrofolate (5-metTHF) serves as a methyl-group (CH_3_) donor in the detoxification of proatherogenic homocysteine to the AA methionine. SAM is the universal CH_3_ donor in histone- and DNA-methylation. This function gives folate powerful mediating properties at an epigenetic level with a potential role in thymic CD4^+^ n*T*_reg_ expansion. Abbreviations: MTR: methionine synthase requiring the co-factor vitamin B12 (cobalamin) as a methyl transfer vehicle (methyl cobalamin, CH_3_-B12); MTHFR: 5,10-methylenetetrahydrofolate reductase (requiring the co-factor NADPH, not shown); THF: tetrahydrofolate. Methionine cycle metabolites: SAH: S-adenosylhomocysteine; SAM: S-adenosylmethionine; =NH: amines.

At a molecular level, folate stabilizes overall cell proliferation, controls DNA modification (histone methylation), and metabolically detoxifies the prooxidative AA intermediate homocysteine, by recycling it to the essential sulphur-rich AA methionine (Figure 3) or alternatively, the semiessential AA cysteine (not depicted in Figure 3).

Folate is delivered to cells through three known routes: (1) via folate receptors (FR)/folate-binding protein (Folbps) [122], (2) the reduced folate carrier, and (3) through the proton-coupled folate transporter [123]. In humans, there are four FR isoforms, namely, *α*, *β*, *γ*, and *δ*, with tissue-specific expression patterns [122, 124]. Initially, three FR isoforms with greater than 70% homology were identified in humans (i.e., *α*, *β*, and *γ*) and two in mice (i.e., *α* and *β*) [125]. The human receptor homologue for murine FR*δ* (also known as FR4 or folate binding protein 3) is expressed on splenic and thymic lymphocytes [126] and is particularly abundant on both n*T*_reg_ and p*T*_reg_, in both mice and humans [65, 127, 128]. Since FR4 is a glycosyl phosphatidylinositol–anchored protein, adapter molecules may assist the receptor in the maintenance of *T*_reg_ cell survival [129, 130] but little is known about its precise role. Importantly, based on its *T*_reg_-specific expression, FR4 can be used to discriminate *T*_reg_ from *T*_conv_ following antigen stimulation [65].

The high folate requirement of murine n*T*_reg_ is met via upregulation of the FR4 surface expression, under the control of FoxP3 [65], suggesting a tight crosstalk between the transcription factor and receptor expression. Folate may also influence other *T*_reg_ molecular pathways (summarised in [114]).

## 7. The Microbiome in Uveitis

Uveitis (intraocular inflammation) is an AD/AID which causes significant blindness and visual handicap worldwide (10–15% in the developed world) [131]. A failure of *T*_reg_ as an underlying pathogenesis is suggested by the reduced numbers of circulating *T*_reg_ in patients with uveitis, and since the number of circulating *T*_reg_ correlates with certain taxa in the colonic microbiome and become stabilized in vivo by bacterial metabolites ([61, 63] see above), this supports a role for a dysregulated microbiome in uveitis. Uveitis occurs in two broad forms, anterior uveitis involving the iris and ciliary body and is closely linked to ankylosing spondylitis (AS) in many cases, and posterior uveitis involving the retina which is protected by the blood retinal barrier (BRB). Both forms of uveitis are subject to changes in the microbiome, particularly anterior uveitis, in conjunction with AS and IBD [132]. Specific autoantigens for human uveitis have been intensively sought but not identified (reviewed in [7]).

Recently, an experimental model of spontaneous uveitis (experimental autoimmune uveoretinitis, EAU) in a transgenic TCR mouse with specificity for a retinal protein (potential autoantigen: interphotoreceptor retinol binding protein, IRBP), in which the mice next to uveitis also develop dysbiosis, has been described. It was suggested that the pathogenic antigen was an unidentified commensal protein which was crossreactive with the IRBP-TCR and, due to the loss of colonic IP (leaky gut), bacterial forms translocated across the gut wall and activated T cells in the gut draining lymph node. Included in this T cell population were autoreactive IRBP-specific T cells which in this mouse model are in increased frequency (~20%) [133]. Once activated, circulating T cells crossed the BRB and were further activated on contact with cognate antigen in the retina causing uveitis and retinal damage. The definitive proof-of-principle experiment was that no uveitis occurred in germ free IRBP-TCR specific mice, i.e., animals lacking a microbiome. Whether the commensal antigen translocated freely in lymphatics or was carried as cargo by trafficking antigen presenting cells is not clear, but trafficking of leukocytes to and from the gut occurs in both health and disease [85, 86], emphasizing a tight immunological crosstalk between the intestine and extraintestinal tissues. While an interesting hypothesis, a similar prevention of EAU was shown in germ free mice [26, 134] in which EAU was induced using a standard procedure of IRBP peptide emulsified in Complete Freund's Adjuvant (CFA) [135]. In this model, disease is induced by a specific antigen in IRBP-specific T cells, in which the precursor frequency of antigen-specific T cells is vanishingly low. In this case, the effect of the microbiome on the induction of uveitis is more likely to be indirect. In another model, in which EAU develops spontaneously due to lymphopenia and imbalance in [*T*_eff_:*T*_reg_] ratio, we have shown that disease can be prevented by adoptive transfer of antigen-experienced *T*_reg_, but not by naïve *T*_reg_. Furthermore, there was evidence of *T*_an_ to *T*_reg_ conversion [90].

It is therefore relatively unexplained how the microbiome influences susceptibility to uveitis and in the context of this review, what might be the role of folate? Recent studies (reviewed in [136]) proposed that EAU in mice might be mediated through epigenetic changes possibly involving Tbx21 and Rorc—two transcription factors important for the differentiation of *T*_reg_ and Th17 cells [137]. Interestingly, hypomethylation of these factors (along with FoxP3) was found in the retinas and RPE-choroidal tissues of B10.RIII mice developing CFA-induced EAU after IRBP immunization, together with an increase in proinflammatory IFN*γ* and IL17 and reduced DNA-methyltransferase 1 (DNMT1) expression in these tissues corresponding to the genes' methylation status ([138]; reviewed in [136]). These findings highlight a requirement for folate to modulate inflammation at an epigenetic level in the prevention of AD and although not stated in that paper, may be linked to the interconversion of *T*_an_ to generate stable *T*_reg_, all under the control of FoxP3. In a separate study, upregulation of miRNA-223 was detected in IRBP-specific Th17 cells from an induced EAU mouse model [139] as well as in uveitis patients' sera [140]. The latter study revealed a pattern of six miRNAs that were linked to inflammatory signalling cascades, such as MAPK, FOXO, and VEGF. Of those miRNAs highlighted, miRNA-223 stood out, as it not only promoted an inflammatory response through activation of DC and T cells but also hinted at a dysbiotic microbiome [141–143] with reduced colonic folate synthesis/bioavailability. Interestingly, hyperhomocysteinaemia, and its underlying polymorphisms in folate metabolism-associated genes [144], occurs in autoinflammatory (Behçet's) uveitis patients [145] indicating a link for FA in noninfectious uveitis [146].

It is clear thus that in the model of autoimmune uveitis, both experimental and clinical there is a strong association with dysbiosis and dysregulated folate metabolism. There is also a clear deficiency in the *T*_reg_ function and/or numbers. Since folate is required for *T*_reg_ physiology [65] the link between folate, *T*_reg_ and autoimmune uveitis speaks for itself [64, 66, 77]. We propose that folate deficiency as part of a dysfunctional microbiome is part of the backdrop to autoimmune uveitis and probably other AD/AID.

## 8. The Microbiome and Its Metabolites as Therapeutic Intervention

There is much interest in potential therapeutic modulation of AD using microbiome-based metabolites including fecal microbial transplantation (FMT), SCFA, folate, and probiotics.

### 8.1. FMT

FMT has been proposed for treatment of AD but it is as yet unclear whether this approach may have a beneficial or deleterious effect [147]. FMT from patients with autoimmune Vogt-Kayanagi-Harada disease (VKH) exacerbated EAU in mice [148]. To date, there are no studies of FMT in uveitis patients.

### 8.2. SCFA

SCFA have been shown to be reduced in patients with RA and in mice with experimental arthritis and interestingly, treatment of such mice with SCFA induced upregulation of the AhR in regulatory B cells [149]. SCFA such as butyrate and propionate have also been effective in reducing inflammation in experimental models including in EAU [86] and endotoxin-induced uveitis [150] but to date have not been translated to clinical use in uveitis. However, the SCFA propionate has been trialed in patients with MS, and a significant shift in the balance towards *T*_reg_ vs Th1/Th17 cells was observed [151].

### 8.3. Folate

Folate and folate supplementation have also been proposed for therapy of AD. In a focal model of EAE, a novel folate-aminopterin construct (EC2319) was found to be tolerated and provided anti-inflammatory benefit by suppressing CD68^+^ macrophage activity [152]. Similarly, a novel FR-targeted drug EC0746 was found to be effective in the treatment of EAE and EAU [153]. The folate receptor FR*β* is expressed on activated macrophages and has been suggested as a target in AD including RA [154]. However, we suggest here that the preferential expression of FR4 on *T*_reg_ promotes their expansion, particularly of colonic *T*_reg_, which then have the ability to suppress macrophage activity.

### 8.4. Probiotics

Delivery of dietary folate to supplement microbiome-generated folate is also a promising approach and may be incorporated in probiotics [155] in combination with prebiotics [156]. Folate-producing lactic acid bacilli, *Streptococcus (Strep.) thermophilus CRL 808 and Strep. thermophilus CRL 415*, have been shown to prevent intestinal inflammation in experimental models and proposed for the treatment of dysbiosis [157]. Probiotics have been proven to prevent EAU in mice, and delivery of folate-producing probiotics offers a safe and tolerable supplement in the treatment of AD [158].

The mechanism of action of folate is distinctly different from other known vitamin-based immunomodulators such as vitamin A/atRA and D, as well as SCFA. While atRA, cholecalciferol (vitamin D3) and SCFA had been found to enhance the peripheral differentiation of naïve T cells into p*T*_reg_ [72, 116–118], and folate is required during clonal expansion of n*T*_reg_ [66]. Hence, folate exerts its modulatory effects at two levels in vivo, namely, (1) as a mediator of epigenetic control in thymic n*T*_reg_; and (2) as an antiapoptotic signal in induced p*T*_reg_ supporting their survival in the circulation. As adoptive transfer of antigen-experienced *T*_reg_ prevents development of EAU [88, 90], it would be interesting to see whether folate-treated antigen-experienced (or even naïve) *T*_reg_ were more effective in control of AD. This could be combined with SCFA to maximize the differentiation of naϊve T cells to *T*_reg_. Complementary effects of the two metabolites are likely based on their different modes of action.

## 9. Folate Deficiency and Current Therapy for AD

Folate deficiency has strong implications for overall health and may also complicate the management of AD. Methotrexate (Mtx; amethopterin) is routinely used to manage a range of AD including certain types of uveitis. As indicated above (see Figure 2), the drug's effectiveness and toxicity vary among individuals and are likely determined by polymorphisms in folate, pyrimidine, and purine metabolic enzymes [159]. The mechanism of action is presumed to be inhibition of T cell proliferation but overall, the efficacy of Mtx in uveitis is limited [160–163]. This may be due to the drug's competitive antifolate effects. Macrophages are major agents of tissue destruction in AD including uveitis [164, 165] and require high amounts of folate to remain active via high surface expression of the folate receptor FR*β* [166, 167]. Mtx is structurally similar to folate with 1000-fold higher affinity for the enzyme dihydrofolate reductase (DHFR) [168, 169] (Figure 3). It thus starves cells with high folate requirement such as activated macrophages, thereby abrogating the cells' survival and halting disease progression. While this might be of benefit to control tissue-damaging macrophages in noninfectious uveitis [170], there may be a negative side-effect on the *T*_reg_ function. Thus, the action of Mtx is likely to be rather complex having both antiproliferative and anti-inflammatory roles and targeting activated macrophages as well as *T*_reg_. In the event, Mtx seems to be more effective in acute AU (with significant myeloid involvement) than in sight-threatening chronic PU (with Th1 and Th17 *T*_eff_ cells being the main drivers of disease).

An alternative mechanism for the immunosuppressive effect of Mtx and other immunosuppressants has been proposed: in humans, hypomethylation of the TSDR (*T*_reg_-specific demethylated region) is required for the functional stability of peripherally expanding FoxP3^+^ p*T*_reg_ [171] and correlates with the duration of oral immunosuppressive therapy. This indicates that in patients, conventional immunosuppression can induce p*T*_reg_ leading to remission of the disease (reviewed in [172]). This finding is particularly interesting with regard to folate being a mediator of epigenetic programming (Figure 3) and emphasizes the importance of coordinating appropriate treatment regimens with the dynamics and kinetics of disease progression.

## 10. Conclusions

Folate as one of the colonic bacterial fermentation products is a powerful micronutrient with a broad spectrum of well-known functions at various levels. Its importance in ocular health is well established [144–146, 173–179], but its immunomodulatory properties represent an emerging concept for functional *T*_reg_ stabilization. The colon is a significant folate depot [19] that participates in metabolism and contributes to bioavailable folate levels. It is an important immunological organ with “unconventional IP” properties [50] with a core function in oral tolerance [180] and prevention of microbial antigen escape into the general circulation. Pathological structural (“leaky gut”) and/or environment-induced proinflammatory changes (dysbiosis) in the gut therefore jeopardize this role, allowing for the transmigration of commensal antigen into the blood stream. The use of folate- and SCFA-producing microbes has the potential to form the basis for a novel approach to prophylactic control of AD. While emerging data suggests oral probiotics [158, 181] or alternatively FMT [182] might help eliminate dysbiosis, knock-on effects on colonic folate synthesis/bioavailability and its implications for immune cell functioning remain to be explored. As dysbiosis has been found to occur in a range of AD, including uveitis [36, 183, 184], targeted administration of certain beneficial folate-producing bacteria or even direct oral folate treatment in AD merits clinical evaluation as a low-risk effective adjunctive treatment option. A daily oral folate supplementation of 5,000–10,000 *μ*g (i.e., 25–50 times the daily recommendation) is generally well tolerated by healthy, nonpregnant individuals. Neurological side-effects have been reported in cases of pernicious anemia (B12 hypovitaminosis), and interference with intestinal zinc absorption has been demonstrated in animals which is likely irrelevant in humans (reviewed in [185]). Some evidence suggests that long-term folic acid supplementation can promote the progression of preexisting malignant lesions in advanced age [186]. Importantly, as *T*_reg_ phenotypes between mice and humans vary to some degree [120, 121], research is needed to clarify those differences and assess whether those AD-attenuating folate effects observed in mice are equally valid in humans. Regardless, folate is an important and undervalued micronutrient with powerful direct and indirect effects in the organism and a potential regulatory role in autoimmunity and chronic inflammation.

## Data Availability

Original data will be made available upon request.

## References

[1] Ben-Chetrit E., Gattorno M., Gul A. (2018). Consensus proposal for taxonomy and definition of the autoinflammatory diseases (AIDs): a Delphi study. *Annals of the Rheumatic Diseases*.

[2] Ruff W. E., Greiling T. M., Kriegel M. A. (2020). Host-microbiota interactions in immune-mediated diseases. *Nature Reviews Microbiology*.

[3] Choileáin S. N., Kleinewietfeld M., Raddassi K., Hafler D. A., Ruff W. E., Longbrake E. E. (2020). CXCR3+ T cells in multiple sclerosis correlate with reduced diversity of the gut microbiome. *Journal of Translational Autoimmunity*.

[4] Dedrick S., Sundaresh B., Huang Q. (2020). The role of gut microbiota and environmental factors in type 1 diabetes pathogenesis. *Frontiers in Endocrinology*.

[5] Manasson J., Blank R. B., Scher J. U. (2020). The microbiome in rheumatology: where are we and where should we go?. *Annals of the Rheumatic Diseases*.

[6] Vural M., Gilbert B., Üstün I., Caglar S., Finckh A. (2020). Mini-review: human microbiome and rheumatic diseases. *Frontiers in Cellular and Infection Microbiology*.

[7] Forrester J. V., Kuffova L., Dick A. D. (2018). Autoimmunity, autoinflammation, and infection in uveitis. *American Journal of Ophthalmology*.

[8] Schön M. P. (2019). Adaptive and innate immunity in psoriasis and other inflammatory disorders. *Frontiers in Immunology*.

[9] Romano M., Fanelli G., Albany C. J., Giganti G., Lombardi G. (2019). Past, present, and future of regulatory T cell therapy in transplantation and autoimmunity. *Frontiers in Immunology*.

[10] Li M., Zhou X., Zhou L., Yu Z., Fu L., Yang P. (2020). Meta-analysis of changes in the number and proportion of regulatory T cells in patients with ankylosing spondylitis. *BioMed Research International*.

[11] Ratsika A., Codagnone M. C., O’Mahony S., Stanton C., Cryan J. F. (2021). Priming for life: early life nutrition and the microbiota-gut-brain axis. *Nutrients*.

[12] Simeoni U., Armengaud J.-B., Siddeek B., Tolsa J.-F. (2018). Perinatal origins of adult disease. *Neonatology*.

[13] Chow B. W., Gu C. (2017). Gradual suppression of transcytosis governs functional blood-retinal barrier formation. *Neuron*.

[14] Ogura S., Kurata K., Hattori Y. (2017). Sustained inflammation after pericyte depletion induces irreversible blood-retina barrier breakdown. *JCI Insight*.

[15] Sekirov I., Russell S. L., Antunes L. C. M., Finlay B. B. (2010). Gut microbiota in health and disease. *Physiological Reviews*.

[16] Griffiths J. K., Ryan E. T. (2020). *144 - Vitamin Deficiencies, in Hunter's Tropical Medicine and Emerging Infectious Diseases*.

[17] WHO (2004). *Vitamin and mineral requirements in human nutrition*.

[18] Greenberg J. A., Bell S. J., Guan Y., Yu Y.-H. (2011). Folic acid supplementation and pregnancy: more than just neural tube defect prevention. *Reviews in Obstetrics and Gynecology*.

[19] Kok D. E., Steegenga W. T., McKay J. A. (2018). Folate and epigenetics: why we should not forget bacterial biosynthesis. *Epigenomics*.

[20] Blakley R. L. (1987). Nomenclature and symbols for folic acid and related compounds. Recommendations 1986. *European Journal of Biochemistry*.

[21] Ohrvik V. E., Witthoft C. M. (2011). Human Folate Bioavailability. *Nutrients*.

[22] Rosenberg I. H., Miller J. W. (1992). Nutritional factors in physical and cognitive functions of elderly people. *American Journal of Clinical Nutrition*.

[23] Selhub J., Bagley L. C., Miller J., Rosenberg I. H. (2000). B vitamins, homocysteine, and neurocognitive function in the elderly. *The American Journal of Clinical Nutrition*.

[24] Kehoe L., Walton J., Flynn A. (2019). Nutritional challenges for older adults in Europe: current status and future directions. *The Proceedings of the Nutrition Society*.

[25] Horai R., Caspi R. R. (2019). Microbiome and autoimmune uveitis. *Frontiers in Immunology*.

[26] Horai R., Zárate-Bladés C. R., Dillenburg-Pilla P. (2015). Microbiota-dependent activation of an autoreactive T cell receptor provokes autoimmunity in an immunologically privileged site. *Immunity*.

[27] de Oliveira G. L. V., Leite A. Z., Higuchi B. S., Gonzaga M. I., Mariano V. S. (2017). Intestinal dysbiosis and probiotic applications in autoimmune diseases. *Immunology*.

[28] Mu Q., Kirby J., Reilly C. M., Luo X. M. (2017). Leaky gut as a danger signal for autoimmune diseases. *Frontiers in Immunology*.

[29] Bach J.-F. (2018). The hygiene hypothesis in autoimmunity: the role of pathogens and commensals. *Nature Reviews Immunology*.

[30] Levy M., Kolodziejczyk A. A., Thaiss C. A., Elinav E. (2017). Dysbiosis and the immune system. *Nature Reviews Immunology*.

[31] Peterson L. W., Artis D. (2014). Intestinal epithelial cells: regulators of barrier function and immune homeostasis. *Nature Reviews Immunology*.

[32] Gill S. R., Pop M., DeBoy R. T. (2006). Metagenomic analysis of the human distal gut microbiome. *Science*.

[33] Rajilić-Stojanović M., de Vos W. M. (2014). The first 1000 cultured species of the human gastrointestinal microbiota. *FEMS Microbiology Reviews*.

[34] Qin J., Consortium M. H. I. T., Li R. (2010). A human gut microbial gene catalogue established by metagenomic sequencing. *Nature*.

[35] Albenberg L. G., Wu G. D. (2014). Diet and the intestinal microbiome: associations, functions, and implications for health and disease. *Gastroenterology*.

[36] Chakravarthy S. K., Jayasudha R., Prashanthi G. S. (2018). Dysbiosis in the gut bacterial microbiome of patients with uveitis, an inflammatory disease of the eye. *Indian Journal of Microbiology*.

[37] Suceveanu A.-I., Dumitru A., Musat M. (2019). Is a fecal microbiota transplant useful for treating inflammatory bowel disease?. *Human Microbiome [working title]*.

[38] Bordon Y. (2019). A microbial trigger for colitis. *Nature Reviews Immunology*.

[39] Shivaji S. (2019). Connect between gut microbiome and diseases of the human eye. *Journal of Biosciences*.

[40] Wu W.-J. H., Zegarra-Ruiz D. F., Diehl G. E. (2020). Intestinal microbes in autoimmune and inflammatory disease. *Frontiers in Immunology*.

[41] Hofley P., Roarty J., McGinnity G. (1993). Asymptomatic uveitis in children with chronic inflammatory bowel diseases. *Journal of Pediatric Gastroenterology and Nutrition*.

[42] Rychwalski P. J., Cruz O. A., Alanis-Lambreton G., Foy T. M., Kane R. E. (1997). Asymptomatic uveitis in young people with inflammatory bowel disease. *Journal of AAPOS*.

[43] Yilmaz S., Aydemir E., Maden A., Unsal B. (2007). The prevalence of ocular involvement in patients with inflammatory bowel disease. *International Journal of Colorectal Disease*.

[44] Edwards F. C., Truelove S. C. (1964). The course and prognosis of ulcerative COLITIS. III. COMPLICATIONS. *Gut*.

[45] Zhao C.-N., Xu Z., Wu G. C. (2019). Emerging role of air pollution in autoimmune diseases. *Autoimmunity Reviews*.

[46] Vojdani A. (2014). A potential link between environmental triggers and autoimmunity. *Autoimmune Diseases*.

[47] Spadoni I., Fornasa G., Rescigno M. (2017). Organ-specific protection mediated by cooperation between vascular and epithelial barriers. *Nature Reviews Immunology*.

[48] Chassaing B., Kumar M., Baker M. T., Singh V., Vijay-Kumar M. (2014). Mammalian gut immunity. *Biomedical Journal*.

[49] Spencer S. P., Fragiadakis G. K., Sonnenburg J. L. (2019). Pursuing human-relevant gut Microbiota-immune interactions. *Immunity*.

[50] Shechter R., London A., Schwartz M. (2013). Orchestrated leukocyte recruitment to immune-privileged sites: absolute barriers versus educational gates. *Nature Reviews Immunology*.

[51] Mölzer C., Heissigerova J., Wilson H. M., Kuffova L., Forrester J. V. (2021). Immune privilege: the microbiome and uveitis. *Frontiers in Immunology*.

[52] Lee B., Moon K. M., Kim C. Y. (2018). Tight junction in the intestinal epithelium: its association with diseases and regulation by phytochemicals. *Journal of Immunology Research*.

[53] Daneman R., Rescigno M. (2009). The gut immune barrier and the blood-brain barrier: are they so different?. *Immunity*.

[54] Ley R. E., Peterson D. A., Gordon J. I. (2006). Ecological and evolutionary forces shaping microbial diversity in the human intestine. *Cell*.

[55] Spadoni I., Zagato E., Bertocchi A. (2015). A gut-vascular barrier controls the systemic dissemination of bacteria. *Science*.

[56] Bush T. G., Savidge T. C., Freeman T. C. (1998). Fulminant jejuno-ileitis following ablation of enteric glia in adult transgenic mice. *Cell*.

[57] Cornet A., Savidge T. C., Cabarrocas J. (2001). Enterocolitis induced by autoimmune targeting of enteric glial cells: a possible mechanism in Crohn's disease?. *Proceedings of the National Academy of Sciences*.

[58] Erny D., de Angelis A. L. H., Jaitin D. (2015). Host microbiota constantly control maturation and function of microglia in the CNS. *Nature Neuroscience*.

[59] Kabouridis P. S., Lasrado R., McCallum S. (2015). Microbiota controls the homeostasis of glial cells in the gut lamina propria. *Neuron*.

[60] Stappenbeck T. S., Hooper L. V., Gordon J. I. (2002). Developmental regulation of intestinal angiogenesis by indigenous microbes via Paneth cells. *Proceedings of the National Academy of Sciences*.

[61] Atarashi K., Tanoue T., Shima T. (2011). Induction of colonic regulatory T cells by indigenous clostridium species. *Science*.

[62] Furusawa Y., Obata Y., Fukuda S. (2013). Commensal microbe-derived butyrate induces the differentiation of colonic regulatory T cells. *Nature*.

[63] Atarashi K., Tanoue T., Oshima K. (2013). Treg induction by a rationally selected mixture of clostridia strains from the human microbiota. *Nature*.

[64] Kinoshita M., Kayama H., Kusu T. (2012). Dietary folic acid promotes survival of Foxp3+ regulatory T cells in the colon. *The Journal of Immunology*.

[65] Yamaguchi T., Hirota K., Nagahama K. (2007). Control of immune responses by antigen-specific regulatory T cells expressing the folate receptor. *Immunity*.

[66] Kunisawa J., Hashimoto E., Ishikawa I., Kiyono H. (2012). A pivotal role of vitamin B9 in the maintenance of regulatory T cells in vitro and in vivo. *PLoS One*.

[67] Rossi M., Amaretti A., Raimondi S. (2011). Folate production by probiotic bacteria. *Nutrients*.

[68] Mellor A. L., Lemos H., Huang L. (2017). Indoleamine 2, 3-dioxygenase and tolerance: where are we now?. *Frontiers in Immunology*.

[69] Zelante T., Iannitti R. . G., Cunha C. (2013). Tryptophan catabolites from microbiota engage aryl hydrocarbon receptor and balance mucosal reactivity via interleukin-22. *Immunity*.

[70] Issazadeh-Navikas S., Teimer R., Bockermann R. (2012). Influence of dietary components on regulatory T cells. *Molecular Medicine (Cambridge, Mass.)*.

[71] Kang S. G., Lim H. W., Andrisani O. M., Broxmeyer H. E., Kim C. H. (2007). Vitamin a metabolites induce gut-homing FoxP3+ regulatory T cells. *The Journal of Immunology*.

[72] Mucida D., Park Y., Kim G. (2007). Reciprocal TH17 and regulatory T cell differentiation mediated by retinoic acid. *Science*.

[73] Xiao S., Jin H., Korn T. (2008). Retinoic acid increases Foxp3+ regulatory T cells and inhibits development of Th17 cells by enhancing TGF-*β*-driven Smad3 signaling and inhibiting IL-6 and IL-23 receptor expression. *The Journal of Immunology*.

[74] Raverdeau M., Christofi M., Malara A. (2019). Retinoic acid-induced autoantigen-specific type 1 regulatory T cells suppress autoimmunity. *EMBO Reports*.

[75] Lavelle A., Sokol H. (2020). Gut microbiota-derived metabolites as key actors in inflammatory bowel disease. *Nature Reviews Gastroenterology*.

[76] den Besten G., van Eunen K., Groen A. K., Venema K., Reijngoud D. J., Bakker B. M. (2013). The role of short-chain fatty acids in the interplay between diet, gut microbiota, and host energy metabolism. *Journal of Lipid Research*.

[77] Trujillo-Vargas C. M., Schaefer L., Alam J., Pflugfelder S. C., Britton R. A., de Paiva C. S. (2020). The gut-eye-lacrimal gland-microbiome axis in Sjogren syndrome. *The Ocular Surface*.

[78] Arpaia N., Campbell C., Fan X. (2013). Metabolites produced by commensal bacteria promote peripheral regulatory T-cell generation. *Nature*.

[79] Mariño E., Richards J. L., McLeod K. H. (2017). Gut microbial metabolites limit the frequency of autoimmune T cells and protect against type 1 diabetes. *Nature Immunology*.

[80] Gong J., Qiu W., Zeng Q. (2019). Lack of short-chain fatty acids and overgrowth of opportunistic pathogens define dysbiosis of neuromyelitis optica spectrum disorders: a Chinese pilot study. *Multiple Sclerosis Journal*.

[81] Zeng Q., Junli Gong, Liu X. (2019). Gut dysbiosis and lack of short chain fatty acids in a Chinese cohort of patients with multiple sclerosis. *Neurochemistry International*.

[82] Blad C. C., Tang C., Offermanns S. (2012). G protein-coupled receptors for energy metabolites as new therapeutic targets. *Nature Reviews Drug Discovery*.

[83] Singh N., Gurav A., Sivaprakasam S. (2014). Activation of Gpr109a, receptor for niacin and the commensal metabolite butyrate, suppresses colonic inflammation and carcinogenesis. *Immunity*.

[84] Engevik M. A., Morra C. N., Röth D. (2019). Microbial metabolic capacity for intestinal folate production and modulation of host folate receptors. *Frontiers in Microbiology*.

[85] Morton A. M., Sefik E., Upadhyay R., Weissleder R., Benoist C., Mathis D. (2014). Endoscopic photoconversion reveals unexpectedly broad leukocyte trafficking to and from the gut. *Proceedings of the National Academy of Sciences*.

[86] Nakamura Y. K., Janowitz C., Metea C. (2017). Short chain fatty acids ameliorate immune-mediated uveitis partially by altering migration of lymphocytes from the intestine. *Scientific Reports*.

[87] Liu S. Q., Alkema P. K., Tieché C. (2005). Negative regulation of monocyte adhesion to arterial elastic laminae by signal regulatory protein *α* and Src homology 2 domain-containing protein-tyrosine phosphatase-1. *The Journal of Biological Chemistry*.

[88] Hara Y., Caspi R. R., Wiggert B., Chan C. C., Wilbanks G. A., Streilein J. W. (1992). Suppression of experimental autoimmune uveitis in mice by induction of anterior chamber-associated immune deviation with interphotoreceptor retinoid-binding protein. *The Journal of Immunology*.

[89] Lambe T., Leung J. C. H., Ferry H. (2007). Limited peripheral T cell anergy predisposes to retinal autoimmunity. *The Journal of Immunology*.

[90] Liu Y.-H., Mölzer C., Makinen K. (2020). Treatment with FoxP3+ antigen-experienced T regulatory cells arrests progressive retinal damage in a spontaneous model of uveitis. *Frontiers in Immunology*.

[91] Prendergast R. A., Iliff C. E., Coskuncan N. M. (1998). T cell traffic and the inflammatory response in experimental autoimmune uveoretinitis. *Investigative Ophthalmology & Visual Science*.

[92] Caspi R. R., Horai R., Zárate-Bladés C. (2014). Activation of autoreactive T cells by endogenous commensal microflora provokes spontaneous autoimmunity in the immunologically privileged eye. *Investigative Ophthalmology & Visual Science*.

[93] Kaisar M. M. M., Pelgrom L. R., van der Ham A. J., Yazdanbakhsh M., Everts B. (2017). Butyrate Conditions Human Dendritic Cells to Prime Type 1 Regulatory T Cells via both Histone Deacetylase Inhibition and G Protein-Coupled Receptor 109A Signaling. *Frontiers in Immunology*.

[94] Luo A., Leach S. T., Barres R., Hesson L. B., Grimm M. C., Simar D. (2017). The microbiota and epigenetic regulation of T Helper 17/regulatory T cells: in search of a balanced immune system. *Frontiers in Immunology*.

[95] Fukuda S., Toh H., Hase K. (2011). Bifidobacteria can protect from enteropathogenic infection through production of acetate. *Nature*.

[96] Macia L., Tan J., Vieira A. T. (2015). Metabolite-sensing receptors GPR43 and GPR109A facilitate dietary fibre- induced gut homeostasis through regulation of the inflammasome. *Nature Communications*.

[97] Johnson E. L., Heaver S. L., Walters W. A., Ley R. E. (2017). Microbiome and metabolic disease: revisiting the bacterial phylum Bacteroidetes. *Journal of Molecular Medicine (Berlin, Germany)*.

[98] Putnam E. E., Goodman A. L. (2020). B vitamin acquisition by gut commensal bacteria. *PLoS Pathogens*.

[99] Ichihashi T., Takagishi Y., Uchida K., Yamada H. (1992). Colonic absorption of Menaquinone-4 and Menaquinone-9 in rats. *The Journal of Nutrition*.

[100] Said H. M., Mohammed Z. M. (2006). Intestinal absorption of water-soluble vitamins: an update. *Current Opinion in Gastroenterology*.

[101] Aufreiter S., Gregory J. F., Pfeiffer C. M. (2009). Folate is absorbed across the colon of adults: evidence from cecal infusion of 13C-labeled [6S]-5-formyltetrahydrofolic acid. *The American Journal of Clinical Nutrition*.

[102] Lakoff A., Fazili Z., Aufreiter S. (2014). Folate is absorbed across the human colon: evidence by using enteric-coated caplets containing 13C-labeled [6S]-5-formyltetrahydrofolate. *The American Journal of Clinical Nutrition*.

[103] Awuchi C., Victory I., Ikechukwu A. (2020). Nutritional diseases and nutrient toxicities: a systematic review of the diets and nutrition for prevention and treatment. *International Journal of Advanced Academic Research*.

[104] Chappert P., Schwartz R. H. (2010). Induction of T cell anergy: integration of environmental cues and infectious tolerance. *Current Opinion in Immunology*.

[105] Kalekar L. A., Mueller D. L. (2017). Relationship between CD4 regulatory T cells and Anergy in vivo. *The Journal of Immunology*.

[106] Mueller D. L. (2010). Mechanisms maintaining peripheral tolerance. *Nature Immunology*.

[107] Sakaguchi S., Yamaguchi T., Nomura T., Ono M. (2008). Regulatory T cells and immune tolerance. *Cell*.

[108] Martinez R. J., Zhang N., Thomas S. R. (2012). Arthritogenic self-reactive CD4+T cells acquire an FR4hiCD73hianergic state in the presence of Foxp3+regulatory T cells. *The Journal of Immunology*.

[109] Morales M. S., Mueller D. (2018). Anergy into T regulatory cells: an integration of metabolic cues and epigenetic changes at the Foxp3 conserved non-coding sequence 2. *F1000Research*.

[110] Kalekar L. A., Schmiel S. E., Nandiwada S. L. (2016). CD4^+^ T cell anergy prevents autoimmunity and generates regulatory T cell precursors. *Nature Immunology*.

[111] Zheng Y., Josefowicz S., Chaudhry A., Peng X. P., Forbush K., Rudensky A. Y. (2010). Role of conserved non-coding DNA elements in the _Foxp3_ gene in regulatory T-cell fate. *Nature*.

[112] Li X., Liang Y., LeBlanc M., Benner C., Zheng Y. (2014). Function of a Foxp3 _cis_ -element in protecting regulatory T cell identity. *Cell*.

[113] Zhang Z., Zhou X. (2019). Foxp3 Instability Helps tTregs Distinguish Self and Non-self. *Frontiers in Immunology*.

[114] Schmidl C., Klug M., Boeld T. J. (2009). Lineage-specific DNA methylation in T cells correlates with histone methylation and enhancer activity. *Genome Research*.

[115] Yoshioka Y., Kozaki T., Ishii K., Toyoda A., Hattori M., Yoshida T. (2017). Comprehensive analysis of epigenetically regulated genes in anergic T cells. *Cellular Immunology*.

[116] Coombes J. L., Siddiqui K. R. R., Arancibia-Cárcamo C. V. (2007). A functionally specialized population of mucosal CD103+ DCs induces Foxp3+ regulatory T cells via a TGF-*β*–and retinoic acid–dependent mechanism. *Journal of Experimental Medicine*.

[117] Sun C.-M., Hall J. A., Blank R. B. (2007). Small intestine lamina propria dendritic cells promote de novo generation of Foxp3 T reg cells via retinoic acid. *The Journal of Experimental Medicine*.

[118] Benson M. J., Pino-Lagos K., Rosemblatt M., Noelle R. J. (2007). All-trans retinoic acid mediates enhanced T reg cell growth, differentiation, and gut homing in the face of high levels of co-stimulation. *The Journal of Experimental Medicine*.

[119] Piantoni S., Andreoli L., Scarsi M. (2015). Phenotype modifications of T-cells and their shift toward a Th2 response in patients with systemic lupus erythematosus supplemented with different monthly regimens of vitamin D. *Lupus*.

[120] Morgan M. E., van Bilsen J. H. M., Bakker A. M. (2005). Expression of FOXP3 mRNA is not confined to CD4^+^CD25^+^ T regulatory cells in humans. *Human Immunology*.

[121] Miyao T., Floess S., Setoguchi R. (2012). Plasticity of Foxp3+ T cells reflects promiscuous Foxp3 expression in conventional T cells but not reprogramming of regulatory T cells. *Immunity*.

[122] Elnakat H., Ratnam M. (2004). Distribution, functionality and gene regulation of folate receptor isoforms: implications in targeted therapy. *Advanced Drug Delivery Reviews*.

[123] Zhao R., Qiu A., Tsai E., Jansen M., Akabas M. H., Goldman I. D. (2008). The proton-coupled folate transporter: impact on pemetrexed transport and on antifolates activities compared with the reduced folate carrier. *Molecular Pharmacology*.

[124] Weitman S. D., Weinberg A. G., Coney L. R., Zurawski V. R., Jennings D. S., Kamen B. A. (1992). Cellular localization of the folate receptor: potential role in drug toxicity and folate homeostasis. *Cancer Research*.

[125] Patrick T. A., Kranz D. M., Dyke T. A. ., Roy E. J., Roy E. J., Roy E. J. (1997). Folate receptors as potential therapeutic targets in choroid plexus tumors of SV40 transgenic mice. *Journal of Neuro-Oncology*.

[126] Spiegelstein O., Eudy J. D., Finnell R. H. (2000). Identification of two putative novel folate receptor genes in humans and mouse. *Gene*.

[127] Walker L. S. (2007). Regulatory T cells: folate receptor 4: a new handle on regulation and memory?. *Journal of Immunology, cell biology*.

[128] Tian Y., Wu G., Xing J. C. (2012). A novel splice variant of folate receptor 4 predominantly expressed in regulatory T cells. *BMC Immunology*.

[129] Jia Z., Zhao R., Tian Y. (2009). A novel splice variant of FR4 predominantly expressed in CD4+ CD25+ regulatory T cells. *Immunological Investigations*.

[130] Shen F., Wu M., Ross J. F., Miller D., Ratnam M. (1995). Folate receptor type gamma is primarily a secretory protein due to lack of an efficient signal for glycosylphosphatidylinositol modification: protein characterization and cell type specificity. *Biochemistry*.

[131] Dick A. D., Tundia N., Sorg R. (2016). Risk of ocular complications in patients with noninfectious intermediate uveitis, posterior uveitis, or panuveitis. *Ophthalmology*.

[132] Rosenbaum J. T., Asquith M. (2018). The microbiome and HLA-B27-associated acute anterior uveitis. *Nature Reviews Rheumatology*.

[133] Horai R., Silver P. B., Chen J. (2013). Breakdown of immune privilege and spontaneous autoimmunity in mice expressing a transgenic T cell receptor specific for a retinal autoantigen. *The Journal of Autoimmunity*.

[134] Heissigerova J., Seidler Stangova P., Klimova A. (2016). The Microbiota determines susceptibility to experimental autoimmune uveoretinitis. *Journal of Immunology Research*.

[135] Huang X. F., Li Z., de Guzman E. (2020). Genomewide association study of acute anterior uveitis identifies new susceptibility loci. *Investigative Ophthalmology & Visual Science*.

[136] Nayyar A., Gindina S., Barron A., Hu Y., Danias J. (2020). Do epigenetic changes caused by commensal microbiota contribute to development of ocular disease? A review of evidence. *Human Genomics*.

[137] Fang D., Zhu J. (2017). Dynamic balance between master transcription factors determines the fates and functions of CD4 T cell and innate lymphoid cell subsets. *Journal of Experimental Medicine*.

[138] Qiu Y., Zhu Y., Yu H., Zhou C., Kijlstra A., Yang P. (2018). Dynamic DNA methylation changes of Tbx21 and Rorc during experimental autoimmune uveitis in mice. *Mediators of Inflammation*.

[139] Wei Y., Chen S., Sun D. (2019). miR-223-3p promotes autoreactive Th17 cell responses in experimental autoimmune uveitis (EAU) by inhibiting transcription factor FOXO3 expression. *The FASEB Journal*.

[140] Verhagen F. H., Bekker C. P. J., Rossato M. (2018). A disease-associated MicroRNA cluster links inflammatory pathways and an altered composition of leukocyte subsets to noninfectious uveitis. *Investigative Ophthalmology & Visual Science*.

[141] Zhou H., Xiao J., Wu N. (2015). MicroRNA-223 regulates the differentiation and function of intestinal dendritic cells and macrophages by targeting C/EBP*β*. *Cell Reports*.

[142] Neudecker V., Haneklaus M., Jensen O. (2017). Myeloid-derived miR-223 regulates intestinal inflammation via repression of the NLRP3 inflammasome. *Journal of Experimental Medicine*.

[143] Wang H., Chao K., Ng S. C. (2016). Pro-inflammatory miR-223 mediates the cross-talk between the IL23 pathway and the intestinal barrier in inflammatory bowel disease. *Genome Biology*.

[144] Messedi M., Frigui M., Chaabouni K. (2013). Methylenetetrahydrofolate reductase C677T and A1298C polymorphisms and variations of homocysteine concentrations in patients with Behcet's disease. *Gene*.

[145] Elbay A. E., Topalkara A., Elbay A., Erdoğan H., Vural A., Çetin A. B. (2015). Evaluation of serum homocysteine and leptin levels in patients with uveitis. *Turkish Journal of Ophthalmology*.

[146] Sijilmassi O. (2019). Folic acid deficiency and vision: a review. *Graefe's Archive for Clinical and Experimental Ophthalmology*.

[147] Marrs T., Walter J. (2021). Pros and cons: is faecal Microbiota transplantation a safe and efficient treatment option for gut dysbiosis?. *Allergy*.

[148] Ye Z., Wu C., Zhang N. (2020). Altered gut microbiome composition in patients with Vogt-Koyanagi-Harada disease. *Gut Microbes*.

[149] Rosser E. C., Piper C. J. M., Matei D. E. (2020). Microbiota-derived metabolites suppress arthritis by amplifying aryl- hydrocarbon receptor activation in regulatory B cells. *Cell Metabolism*.

[150] Chen N., Wu J., Wang J. (2021). Short chain fatty acids inhibit endotoxin-induced uveitis and inflammatory responses of retinal astrocytes. *Experimental Eye Research*.

[151] Duscha A., Gisevius B., Hirschberg S. (2020). Propionic acid shapes the multiple sclerosis disease course by an immunomodulatory mechanism. *Cell*.

[152] Elo P., Li X. G., Liljenbäck H. (2021). Efficacy and tolerability of folate-aminopterin therapy in a rat focal model of multiple sclerosis. *Journal of Neuroinflammation*.

[153] Lu Y., Wollak K. N., Cross V. A. (2014). Folate receptor-targeted aminopterin therapy is highly effective and specific in experimental models of autoimmune uveitis and autoimmune encephalomyelitis. *Clinical Immunology*.

[154] Nogueira E., Gomes A. C., Preto A., Cavaco-Paulo A. (2016). Folate-targeted nanoparticles for rheumatoid arthritis therapy. *Nanomedicine: Nanotechnology, Biology*.

[155] Evans E., Piccio L., Cross A. H. (2018). Use of vitamins and dietary supplements by patients with multiple sclerosis: a review. *JAMA Neurology*.

[156] Sanders M. E., Merenstein D. J., Reid G., Gibson G. R., Rastall R. A. (2019). Probiotics and prebiotics in intestinal health and disease: from biology to the clinic. *Nature Reviews Gastroenterology*.

[157] de Moreno de LeBlanc A., Levit R., de Giori G. S., LeBlanc J. G. (2018). Vitamin producing lactic acid bacteria as complementary treatments for intestinal inflammation. *Anti-Inflammatory & Anti-Allergy Agents in Medicinal Chemistry*.

[158] Dusek O., Fajstova A., Klimova A. (2021). Severity of experimental autoimmune uveitis is reduced by pretreatment with live probiotic Escherichia coli nissle 1917. *Cell*.

[159] Campalani E., Arenas M., Marinaki A. M., Lewis C. M., Barker J. N. W. N., Smith C. H. (2007). Polymorphisms in folate, pyrimidine, and purine metabolism are associated with efficacy and toxicity of methotrexate in psoriasis. *Journal of Investigative Dermatology*.

[160] Gangaputra S., Newcomb C. W., Liesegang T. L. (2009). Methotrexate for ocular inflammatory diseases. *Ophthalmology*.

[161] Ma C. S., Phan T. G. (2017). Here, there and everywhere: T follicular helper cells on the move. *Immunology*.

[162] Muñoz-Fernández S., Martín-Mola E. (2006). Uveitis. *Best Practice & Research. Clinical Rheumatology*.

[163] Samson C. M., Waheed N., Baltatzis S., Foster C. S. (2001). Methotrexate therapy for chronic noninfectious uveitis: analysis of a case series of 160 patients. *Ophthalmology*.

[164] Chinnery H. R., McMenamin P. G., Dando S. J. (2017). Macrophage physiology in the eye. *Pflügers Archiv - European Journal of Physiology*.

[165] Multicenter Uveitis Steroid Treatment Trial Research Group, Kempen J. H., Altaweel M. M., Holbrook J. T., Jabs D. A., Sugar E. A. (2010). The multicenter uveitis steroid treatment trial: rationale, design, and baseline characteristics. *American Journal of Ophthalmology*.

[166] Nakashima-Matsushita N., Homma T., Yu S. (1999). Selective expression of folate receptor ? and its possible role in methotrexate transport in synovial macrophages from patients with rheumatoid arthritis. *Arthritis & Rheumatology*.

[167] Xia W., Hilgenbrink A. R., Matteson E. L., Lockwood M. B., Cheng J. X., Low P. S. (2009). A functional folate receptor is induced during macrophage activation and can be used to target drugs to activated macrophages. *Blood*.

[168] Pastore E. J., Kisliuk R. L., Plante L. T., Wright J. M., Kaplan N. O. (1974). Conformational changes induced in dihydrofolate reductase by folates, pyridine nucleotide coenzymes, and methotrexate. *Proceedings of the National Academy of Sciences*.

[169] Williams J. W., Morrison J. F., Duggleby R. G. (1979). Methotrexate, a high-affinity pseudosubstrate of dihydrofolate reductase. *Biochemistry*.

[170] Gangaplara A., Massilamany C., Steffen D., Reddy J. (2013). Mimicry epitope from _Ehrlichia canis_ for interphotoreceptor retinoid-binding protein 201 -216 prevents autoimmune uveoretinitis by acting as altered peptide ligand. *Journal of Neuroimmunology*.

[171] Schreiber L., Pietzsch B., Floess S. (2014). The Treg-specific demethylated region stabilizes Foxp3 expression independently of NF-*κ*B signaling. *PLoS One*.

[172] Wildner G., Diedrichs-Möhring M. (2019). Resolution of uveitis. *Seminars in Immunopathology*.

[173] Bleich S., Kornhuber J., Junemann A. G. (2003). Homocysteine in primary and secondary open-angle glaucoma. *Journal of Glaucoma*.

[174] de Silva P., Jayamanne G., Bolton R. (2008). Folic acid deficiency optic neuropathy: a case report. *Journal of Medical Case Reports*.

[175] Huang P., Wang F., Kumar Sah B. (2015). Homocysteine and the risk of age-related macular degeneration: a systematic review and meta-analysis. *Scientific Reports*.

[176] Knox D. L., Chen M. F., Guilarte T. R., Dang C. V., Burnette J. (1982). Nutritional amblyopia. Folic acid, vitamin B-12, and other vitamins. *Retina*.

[177] Moore K. J., Carmichael S. L., Forestieri N. E. (2020). Maternal diet as a risk factor for primary congenital glaucoma and defects of the anterior segment of the eye in the National Birth Defects Prevention Study. *Birth Defects Research*.

[178] Sen S. K., Pukazhvanthen P., Abraham R. (2008). Plasma homocysteine, folate and vitamin B(12) levels in senile cataract. *Indian Journal of Clinical Biochemistry*.

[179] Xu C., Wu Y., Liu G., Liu X., Wang F., Yu J. (2014). Relationship between homocysteine level and diabetic retinopathy: a systematic review and meta-analysis. *Diagnostic Pathology*.

[180] Goubier A., Dubois B., Gheit H. (2008). Plasmacytoid dendritic cells mediate oral tolerance. *Immunity*.

[181] Kim J., Choi S., Kim Y. (2017). Clinical Effect of IRT-5 probiotics on immune modulation of autoimmunity or alloimmunity in the eye. *Nutrients*.

[182] Choi R. Y., Asquith M., Rosenbaum J. T. (2018). Fecal transplants in spondyloarthritis and uveitis: ready for a clinical trial?. *Current Opinion in Rheumatology*.

[183] Huang X., Ye Z., Cao Q. (2018). Gut Microbiota composition and fecal metabolic phenotype in patients with acute anterior uveitis. *Investigative Ophthalmology & Visual Science*.

[184] Jayasudha R., Kalyana Chakravarthy S., Sai Prashanthi G., Sharma S., Tyagi M., Shivaji S. (2019). Implicating dysbiosis of the gut fungal microbiome in uveitis, an inflammatory disease of the eye. *Investigative Ophthalmology & Visual Science*.

[185] Butterworth C. E., Tamura T. (1989). Folic acid safety and toxicity: a brief review. *The American Journal of Clinical Nutrition*.

[186] Wien T. N., Pike E., Wisløff T., Staff A., Smeland S., Klemp M. (2012). Cancer risk with folic acid supplements: a systematic review and meta-analysis. *BMJ Open*.

